# Epidemiological changes and outcomes of people living with HIV admitted to the intensive care unit: a 14-year retrospective study

**DOI:** 10.1007/s15010-024-02402-x

**Published:** 2024-10-11

**Authors:** Esther Martínez, Alberto Foncillas, Adrián Téllez, Sara Fernández, Gemma Martínez-Nadal, Verónica Rico, Adrià Tomé, Ainoa Ugarte, Mariano Rinaudo, Leire Berrocal, Elisa De Lazzari, Jose M. Miró, Jose M. Nicolás, Josep Mallolas, Lorena De la Mora, Pedro Castro

**Affiliations:** 1 Intensive Care Unit, Hospital General de Granollers, Granollers, Spain; 2 Intensive Care Unit, Hospital Sant Joan Despí Moisès Broggi, Sant Joan Despí, Spain; 3 University of Barcelona, Barcelona, Spain; 4 Infectious Diseases Department, Hospital Clínic, Barcelona, Spain; 5 Medical Intensive Care Unit, Hospital Clínic, Barcelona, Spain; 6 Emergency Department, Hospital Clínic, Barcelona, Spain; 7 Hospital at Home, Hospital Clínic, Barcelona, Spain; 8 Emergency Department, Complejo Hospitalario Universitario A Coruña, A Coruña, Spain; 9 Intensive Care Unit, Hospital Universitari de Vic, Vic, Spain; 10 IDIBAPS, Barcelona, Spain; 11 CIBERINFEC, Instituto de Salud Carlos III, Madrid, Spain

**Keywords:** Intensive care unit (ICU), Human immunodeficiency virus (HIV), HIV infection, Critical care, Antiretroviral therapy (ART), Acquired immunodeficiency syndrome (AIDS)

## Abstract

**Purposes:**

Since 2016, the World Health Organization has recommended universal antiretroviral therapy (ART) for all people living with Human Immunodeficiency Virus (PLHIV). This recommendation may have influenced the characteristics and outcomes of PLHIV admitted to the Intensive Care Unit (ICU). This study aims to identify changes in the epidemiological and clinical characteristics of PLHIV admitted to the ICU, and their short- and medium-term outcomes before and after the implementation of universal ART (periods 2006–2015 and 2016–2019).

**Methods:**

This retrospective, observational, single-center study included all adult PLHIV admitted to the ICU of a University Hospital in Barcelona from 2006 to 2019.

**Results:**

The study included 502 admissions involving 428 patients, predominantly men (75%) with a median (P25-P75) age of 47.5 years (39.7–53.9). Ninety-one percent were diagnosed with HIV before admission, with 82% under ART and 60% admitted from the emergency department. In 2016–2019, there were more patients on ART pre-admission, reduced needs for invasive mechanical ventilation (IMV) and fewer in-ICU complications. ICU mortality was also lower (14% vs 7%). Predictors of in-ICU mortality included acquired immunodeficiency syndrome defining event (ADE)-related admissions, ICU complications, higher SOFA scores, IMV and renal replacement therapy (RRT) requirement. ART use during ICU admission was protective. Higher SOFA scores, admission from hospital wards, and more comorbidities predicted one-year mortality.

**Conclusions:**

The in-ICU mortality of critically ill PLHIV has decreased in recent years, likely due to changes in patient characteristics. Pre- and ICU admission features remain the primary predictors of short- and medium-term outcomes.

**Supplementary Information:**

The online version contains supplementary material available at 10.1007/s15010-024-02402-x.

## Introduction

The epidemiology and prognosis of people living with Human Immunodeficiency Virus (PLHIV) have substantially improved over the last two decades, thanks to more potent and safer antiretroviral therapy (ART) [1, 2]. These changes are also evident in PLHIV requiring Intensive Care Unit (ICU) admission, who now have significantly better short- and long-term survival rates compared to the early years of the epidemic, when their prognosis was often considered fatal, and ICU admission was deemed inappropriate [3–5].

The improved prognosis for these patients is largely due to advancements in critical illness management and, most importantly, enhancements in HIV treatment. The availability and efficacy of ART, along with optimized virological suppression and immunological recovery, have led to changes in the reasons for ICU admission, decreasing those related to acquired immunodeficiency syndrome (AIDS)-defining events (ADE), including opportunistic infections (OI), and increasing non-ADE admissions [4, 6–10].

Following several clinical trials demonstrating the benefits of early ART initiation [11, 12], the World Health Organization (WHO) recommended early ART for all PLHIV in 2016, regardless of immunological status [13]. The impact of this recommendation on critically ill PLHIV, particularly concerning ART use during and after ICU admission, as well as short- and medium-term outcomes and prognosis, remains largely unknown.

This study aims to identify any changes in the epidemiological and clinical characteristics of PLHIV admitted to the ICU and their short- and medium-term outcomes before and after the implementation of universal ART in 2016.

## Materials and methods

This article was written following STROBE recommendations.

### Design, setting, and study population

This retrospective, observational, single-center study included all consecutive ICU admissions at Hospital Clinic of Barcelona for more than 12 h, with an HIV diagnosis made before, during, or immediately after ICU admission. Exclusions were made for patients admitted only to resuscitation areas or emergency boxes, as well as ICU readmissions during the same hospitalization or within one-month post-ICU discharge. The study period covered from 17/11/2006 to 31/12/2019. To identify temporal changes, ICU admissions were divided into two periods: 2006–2015 and 2016–2019, with the latter period marking the WHO’s universal ART recommendation. Data were sourced from electronic medical records.

### Study variables

Variables included: i. general information [demographics, medical history]; ii. HIV infection-related information [diagnosis date, pre-admission and ICU plasma RNA viral load (VL), CD4 + T-lymphocytes counts (absolute number and percentage, nadir, pre-admission, and in-ICU), previous ADE (including OI), ART regimens, and adherence]; iii. ICU admission-related information [admission date, cause, vital signs and main laboratory values at admission, 24 h, 48 h and 72 h, Acute Physiology And Chronic Health Evaluation (APACHE) II and Sequential Organ Failure Assessment (SOFA) scores at admission, need for mechanical ventilation (MV), renal replacement therapy (RRT), or vasopressors, complications, ICU mortality, ART use, and discharge date]; and iv. outcomes at ICU discharge [status at 3-, 6-, and 12-months post- discharge, ART use, immunological status, and mortality].

When assessing comorbidities, distinctions were made between those related to toxic habits (smoking, alcoholism, intravenous drug use, Hepatitis C Virus infection (HCV), Hepatitis B Virus infection and liver cirrhosis) and unrelated comorbidities. Detailed variable definitions and measurement methods are provided in supplementary information (SI1).

### Statistical analysis

Quantitative variables were described using medians and interquartile ranges. Qualitative variables were described by frequencies and absolute percentages. To compare admissions between the two periods (2006–2015 vs. 2016–2019) and to identify risk factors for ICU mortality and one-year mortality post-ICU discharge (response variables), univariable logistic regressions were conducted with each of the 3 response variables and each variable of interest as a covariate. For each response variable, multiple logistic regression was performed with variables found statistically significant in the univariable analysis as covariates, alongside clinically significant variables. Final models were selected using a backward stepwise variable selection approach. For ICU mortality, all admissions were considered, and all variables collected before ICU discharge or in-ICU decease were evaluated. For one-year mortality, only admissions discharged alive from ICU and variables after discharge were considered. Kaplan–Meier curves depicted survival probabilities. For ICU mortality, time from admission to in-ICU death was evaluated. Those admissions not presenting the outcome were censored at the time of ICU discharge. For one-year after ICU discharge mortality, time from ICU discharge to death was evaluated. Discharged admissions were censored if they were readmitted, were lost to follow up before 1 year, or at 365 days. Robust standard errors specifying patients as clusters were calculated for both univariable and multivariable logistic regression models. Both univariable and multivariable logistic regression models were adjusted by sex and age at ICU admission. All tests were two-tailed, and the statistical significance threshold was set to 5%, except when selecting statistically significant covariates from the univariable logistic regressions to add them as covariates in the multivariable logistic regression models, where statistical significance threshold was set to 10%. Statistical analyses were conducted using Stata [14].

### Ethics

The project (Evolution of HIV patients’ epidemiology in the ICU 2000–2020) was approved by the Institutional Review Board of the Hospital Clinic of Barcelona (Reference HCB/2023/0077) on February 9th, 2023.

## Results

### Characteristics and outcomes

The study included 502 admissions involving 428 patients. Baseline characteristics are summarized in Table 1 and ST1. The majority were male (75%), with a median (P25-P75) age of 47.5 years (39.7–53.9). Ninety-one percent were diagnosed with HIV before ICU admission, with a median duration since diagnosis of 15.7 years (7.4–21.7).

**Table 1:** Main characteristics of admissions included in the study (total and by periods, univariable analysis)

Variables^*a*^	Total (n = 502)	2006–2015 (n = 328)	2016–2019 (n = 174)	OR (95% CI)	p Value
Sex (male)^b^	375 (75%)	249 (76%)	126 (72%)	–*	–*
Age (years)^b^	47.5 (39.7–53.9)	46.1 (39.6–52.4)	50 (41.5–57.3)	–*	–*
Number of comorbidities	0 (0–2)	0 (0–1)	1 (0–2)	1.09 (0.91–1.31)	0.336
IVDU^c^	179 (36%)	139 (42%)	40 (23%)	0.40 (0.24–0.67)	0.001
Alcohol consumption	139 (28%)	102 (31%)	37 (21%)	0.60 (0.37–0.96)	0.035
HCV-coinfection	226 (45%)	161 (49%)	65 (37%)	0.61 (0.40–0.93)	0.022
Previous HIV diagnosis at admission	459 (91%)	299 (91%)	160 (92%)	0.96 (0.48–1.90)	0.902
Time between HIV diagnosis and Hospital admission (years) (n = 420)	15.71 (7.41–21.68)	13.41 (6.31–19.31)	20.17 (9.77–25.72)	1.08 (1.04–1.11)	< 0.001
Undetectable VL^d^ pre-admission (n = 403)	257 (64%)	156 (60%)	101 (70%)	1.34 (0.85–2.12)	0.205
CD4 + count pre-admission (cells/mm^3^) (n = 398)	254 (102–468)	243 (111–443)	290 (82–536)	1.02 (0.98–1.06)^e^	0.268
ART pre-admission (n = 445)	367 (82%)	230 (79%)	137 (88%)	1.73 (0.92–3.23)	0.087
Pre-admission OI prophylaxis (n = 417)	86 (21%)	62 (23%)	24 (17%)	0.71 (0.42–1.20)	0.202
Admission source					
Emergency department	299 (60%)	191 (58%)	108 (62%)	1	0.632
Hospital ward	111 (22%)	75 (23%)	36 (21%)	0.87 (0.54–1.41)	
Others (operating room, another ICU)	92 (18%)	62 (19%)	30 (17%)	0.79 (0.47–1.32)	
Days of hospitalization before ICU^f^ (n = 111)	8 (4–16)	8 (4–19)	8.5 (3–14.5)	0.98 (0.95–1.02)	0.383
Type of ICU admission (medical/surgical)					
Medical admission	384 (76%)	244 (74%)	140 (80%)	1	0.097
Surgical admission (elective)	76 (15%)	50 (15%)	26 (15%)	0.81 (0.47–1.40)	
Surgical admission (urgent)	42 (8%)	34 (10%)	8 (5%)	0.43 (0.20–0.95)	
Main cause of ICU admission^g^					
Infectious process	273 (54%)	183 (56%)	90 (52%)	0.90 (0.61–1.31)	0.567
Respiratory infection	162 (32%)	121 (37%)	41 (24%)	0.54 (0.35–0.84)	0.006
ADE	109 (22%)	72 (22%)	37 (21%)	1.04 (0.65–1.64)	0.880
OI	94 (19%)	64 (20%)	30 (17%)	0.91 (0.56–1.48)	0.702
Surgical process (elective and non-elective)	121 (24%)	87 (27%)	34 (20%)	0.64 (0.40–1.03)	0.065
APACHE II (admission)	18 (14–22)	18 (14–22)	18 (13–21)	0.97 (0.94–1.00)	0.080
SOFA (admission)	5.5 (3–8)	6 (4–9)	4.5 (3–7)	0.89 (0.84–0.95)	< 0.001
SOFA at 48 h	4 (3–6)	4 (3–7)	4 (2–5)	0.91 (0.85–0.97)	0.006
Need of IMV	216 (43%)	170 (52%)	46 (26%)	0.34 (0.22–0.51)	< 0.001
Need of NIV	68 (14%)	46 (14%)	22 (13%)	0.83 (0.43–1.62)	0.583
Vasopressors	220 (44%)	165 (50%)	55 (32%)	0.46 (0.30–0.69)	< 0.001
Norepinephrine	218 (43%)	164 (50%)	54 (31%)	0.45 (0.30–0.68)	< 0.001
Epinephrine	16 (3%)	10 (3%)	6 (3%)	1.07 (0.39–2.93)	0.889
Dobutamine	30 (6%)	21 (6%)	9 (5%)	0.71 (0.30–1.68)	0.441
RRT	36 (7%)	20 (6%)	16 (9%)	1.76 (0.80–3.86)	0.162
Total parenteral nutrition	90 (18%)	68 (21%)	22 (13%)	0.57 (0.33–0.99)	0.047
ART during ICU admission (n = 429)	228 (53%)	140 (50%)	88 (59%)	1.40 (0.89–2.20)	0.144
For the first time (naïve patient) (n = 21)	14 (7%)	9 (7%)	5 (6%)	0.93 (0.29–3.01))	0.901
VL (copies/mL) in ICU (n = 247)	154 (49–116,800)	166.5 (49–120,500)	135 (49–82,300)	1.00 (1.00–1.00)	0.273
Only in detectable patients (n = 135)	82,300 (6276–336,400)	105,900 (8641–295,600)	68,220 (2010–511000)	1.00 (1.00–1.00)	0.345
CD4 + count (cells/mm^3^) in ICU (n = 233)	109 (37–262)	115 (41–260)	78 (24–281)	0.97 (0.91–1.04)^e^	0.432
Complications in ICU (n = 500)	210 (42%)	160 (49%)	50 (29%)	0.43 (0.29–0.65)	< 0.001
Surgical wound infection	49 (10%)	47 (14%)	2 (1%)	0.07 (0.02–0.29)	< 0.001
UTI	26 (5%)	17 (5%)	9 (5%)	0.90 (0.38–2.13)	0.811
VAP	24 (5%)	19 (6%)	5 (3%)	0.47 (0.17–1.31)	0.150
CRBSI	25 (5%)	21 (6%)	4 (2%)	0.33 (0.11–1.01)	0.052
Non-CRBSI	41 (8%)	38 (12%)	3 (2%)	0.14 (0.04–0.46)	0.001
ICU length of stay (days)	4 (2–9)	4 (2–10)	4 (2–8)	0.99 (0.97–1.01)	0.190
Hospital length of stay (days) (n = 500)	17 (8–33)	18 (9–35)	16 (8–29)	1.00 (0.99–1.01)	0.527
ICU readmission during the same stay (n = 369)	28 (8%)	12 (5%)	16 (11%)	2.07 (0.94–4.56)	0.073
Death during ICU admission	59 (12%)	47 (14%)	12 (7%)	0.45 (0.23–0.88)	0.020
Death during entire hospitalization^h^	88 (18%)	66 (20%)	22 (13%)	0.55 (0.32–0.95)	0.032

Categorical variables are expressed as n (%) and quantitative variables as median (P25-P75)

*ADE* acquired immunodeficiency syndrome (AIDS)-defining event, *APACHE II* acute physiology and chronic health evaluation, *ART* antiretroviral therapy, *CI* confidence interval, *CRBSI* catheter related bloodstream infection, *HCV* hepatitis C virus, *HIV* human immunodeficiency virus, *ICU* intensive care unit, *IMV* invasive mechanical ventilation, *IVDU* intravenous drug users, *NIV* non-invasive ventilation, *OI* opportunistic infection, *OR* odds ratio, *RRT* renal replacement therapy, *SOFA* sepsis-related organ failure assessment, *UTI* urinary tract infection, *VAP* ventilator-associated pneumonia, *VL* viral load (copies/ml)

^*a*^Under each variable, the sample size (n) on which the analysis was conducted is provided if it is not the whole cohort (patients for whom the information was not available are excluded). ^*b*^Used as adjustment variable; ^*c*^Past and current; ^*d*^ < 50 copies/ml; ^*e*^Per increase of 50 cells/mm^3^; ^*f*^Only for patients admitted from hospitalization wards; patients coming from emergency department are excluded; ^*g*^One patient could share several categories; ^*h*^Includes deaths both in ICU and in hospitalization. *These variables are for adjustment, and their odds ratio (OR) are not interpreted

Most admissions (60%, 299/502) were from the emergency department. The most frequent reasons for admission were non-ADE (78%, 393/502). ADE admissions were mostly due to OI (86%, 94/109).

APACHE II and SOFA scores at admission were 18 (14–22) and 5.5 (3–8), respectively. Forty-three percent of patients required invasive mechanical ventilation (IMV), 44% required vasopressors, and 7% needed RRT.

**Fig. 1:**
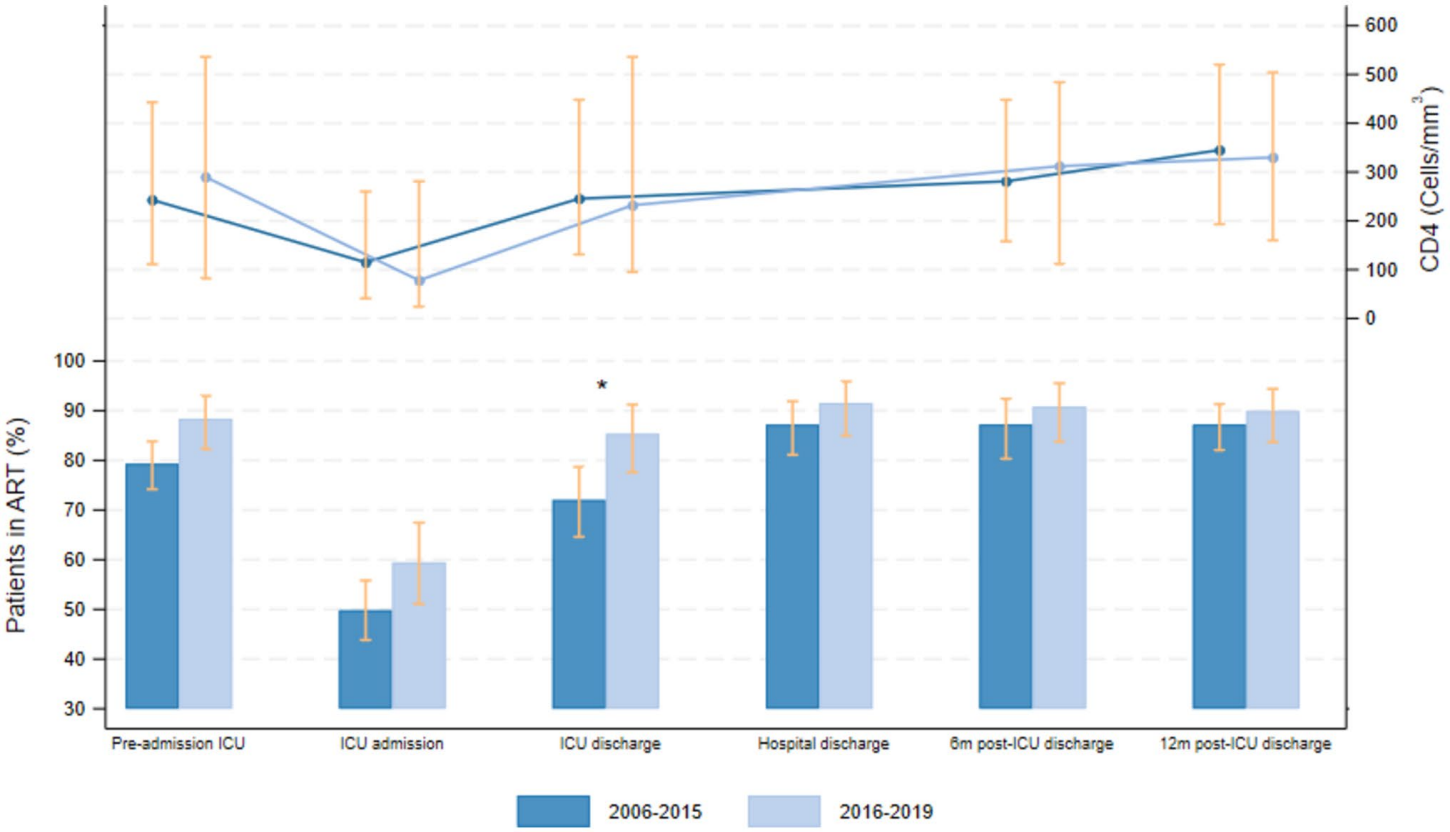
ART use during different periods and CD4 T cell count in total and by periods (2006–2015 and 2016–2019). ART: antiretroviral therapy; ICU: intensive care unit; m: months. *p < 0.05 comparing 2006–2015 and 2016–2019 periods

ART use was prevalent, with 82% of admissions on ART pre-ICU, 53% during ICU stay, and 89–89-88% of survivors continuing ART upon hospital discharge, at 6 and 12 months, respectively (Tables 1, 2; Fig. 1). ICU survivors showed progressive CD4 T cell count increases post-discharge (Table 2; Fig. 1).

**Table 2:** Characteristics related to ICU discharge outcomes of ICU survivors included in the study (total and by periods, univariable analysis)

Variables^*a*^	Total (n = 443)	2006–2015 (n = 281)	2016–2019 (n = 162)	OR (95% CI)	p Value
Sex (male)^b^	326 (74%)	210 (75%)	116 (72%)	–*	–*
Age (years)^b^	47 (39.7–53.9)	45.9 (39.6–51.8)	50.1 (42.2–57.4)	–*	–*
Hospital readmission at 3 months post-ICU discharge (n = 310)	117 (38%)	55 (30%)	62 (49%)	2.21 (1.32–3.70)	0.003
ICU readmission at 3 months (n = 116)	26 (22%)	15 (28%)	11 (18%)	0.58 (0.22–1.54)	0.277
Hospital readmission at 12 months post-ICU discharge (n = 269)	94 (35%)	43 (29%)	51 (42%)	1.89 (1.06–3.39)	0.032
ICU readmission at 12 months (n = 92)	10 (11%)	5 (12%)	5 (10%)	0.59 (0.14–2.42)	0.463
Therapies after ICU discharge:					
Permanent hemodialysis (n = 31)	9 (29%)	4 (27%)	5 (31%)	1.74 (0.24–12.89)	0.586
Home oxygen therapy (n = 352)	17 (5%)	7 (3%)	10 (7%)	2.25 (0.46–11.07)	0.319
Home NIV (n = 355)	2 (0%)	1 (0%)	1 (1%)	1.49 (0.13–17.09)	0.748
Discharge to a Nursing Home (n = 341)	31 (9%)	20 (10%)	11 (8%)	0.73 (0.32–1.68)	0.459
Continued ART at ICU discharge (n = 284)	220 (77%)	121 (72%)	99 (85%)	2.11 (1.12–3.99)	0.021
Continued ART at hospital discharge (n = 282)	251 (89%)	143 (87%)	108 (92%)	1.51 (0.67–3.36)	0.319
Continued ART at 6 months (n = 242)	215 (89%)	116 (87%)	99 (91%)	1.28 (0.53–3.08)	0.585
Continued ART at 12 months (n = 358)	316 (88%)	191 (87%)	125 (90%)	1.38 (0.66–2.90)	0.391
First CD4 count (cells/mm^3^) post-ICU discharge (n = 255)	245 (103–483)	245.5 (131–448)	232 (95–536)	1.03 (0.98–1.08)^c^	0.243
CD4 count (cells/mm^3^) 3–6 months post-ICU discharge (n = 207)	296 (151–464)	281 (158–448)	312 (111.5–484)	1.02 (0.97–1.08)^c^	0.440
CD4 count (cells/mm^3^) 6–12 months post-ICU discharge (n = 212)	332 (186.5–519)	345 (193–520)	330 (160–504)	0.98 (0.92–1.04)^c^	0.483
Death within one-year post-ICU discharge (n = 443)	64 (14%)	44 (16%)	20 (12%)	0.64 (0.36–1.14)	0.133

Categorical variables are expressed as n (%) and quantitative variables as median (P25-P75)

*ART* antiretroviral therapy, *CI* confidence interval, *ICU* intensive care unit, *NIV* non-invasive ventilation, *OR* odds ratio

^*a*^Under each variable, the sample size (n) on which the analysis was conducted is provided (ICU non-survivors and patients for whom the information was not available are excluded); ^*b*^Used as adjustment variable; ^*c*^Per increase of 50 cells/mm^3^. *These variables are for adjustment, and their odds ratio (OR) are not interpreted

Mortality rates were 12% in the ICU and 18% during hospitalization. Among ICU survivors, one-year mortality was 14%.

### Temporal changes in admission characteristics and outcome

**Fig. 2:**
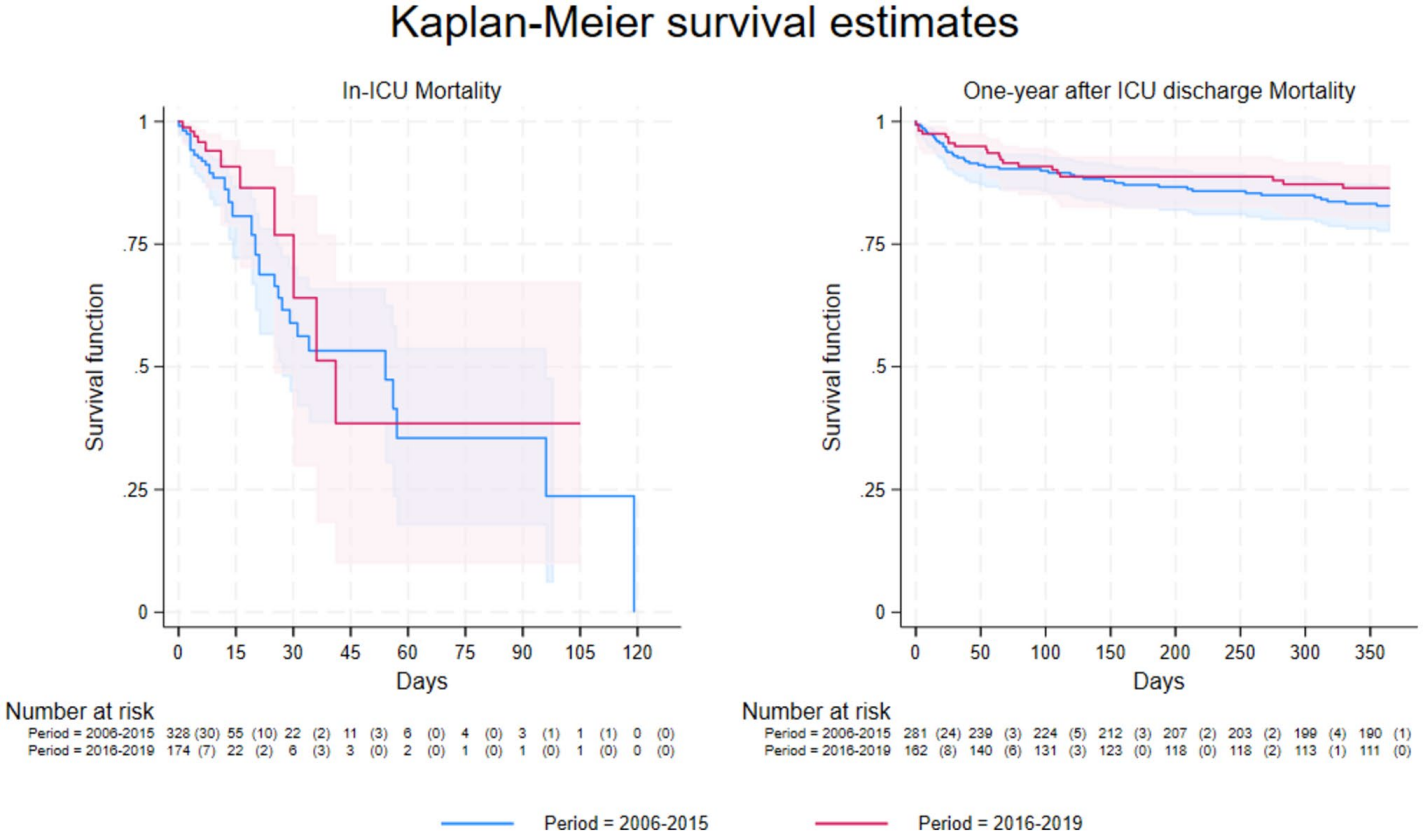
Kaplan–Meier survival curves comparing the periods of study (2006–2015 and 2016–2019). **A** ICU survival probability. **B** One-year survival probability (among ICU survivors). ICU: intensive care unit

Comparing the two periods, significant differences emerged in patients’ characteristics (Tables 1, ST1 and ST2). Mortality was lower in the second period (14% vs. 7% (OR 0.45 [0.23–0.88]; p = 0.020) and one-year post-ICU discharge (16% vs. 12%, OR 0.64 [0.36–1.14], p = 0.133), although the latter did not reach statistical significance (Tables 1, 2). Kaplan–Meier curves illustrate survival probabilities (Fig. 2).

After multivariable adjustment, significant differences persisted in admission characteristics (ST3). The second period saw more patients on ART pre-admission (OR 2.03 [1.00–4.11]; p = 0.049), fewer current/former intravenous drug users (IVDU) (OR 0.41 [0.24–0.72]; p = 0.002), fewer surgical admissions (OR 0,55 [0.32–0.95]; p = 0.033), lower IMV need (OR 0.42 [0.26–0.68]; p < 0.001), and fewer ICU complications such as surgical wound infection (OR 0.04 [0.00–0.28]; p = 0.001) and non-catheter-related bloodstream infection (non-CRBSI) (OR 0.13 [0.03–0.56]; p = 0.006). For ICU survivors, RRT need also increased in the second period (OR 5.74 [1.87–17.66]; p = 0.002). ICU and one-year mortality rates were not significantly different among periods in this adjusted analysis.

### Predictors of ICU and one-year mortality

**Fig. 3:**
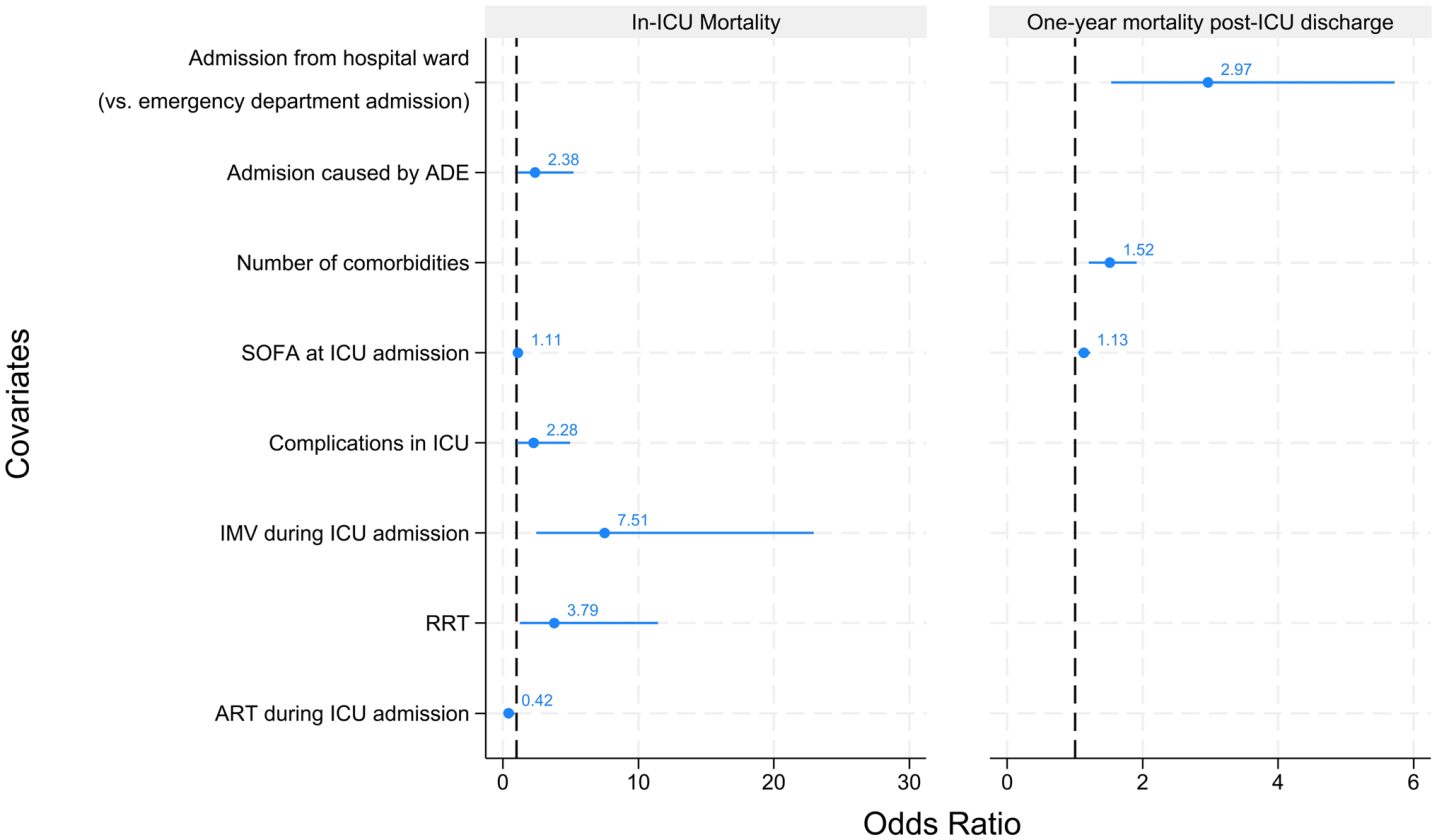
Prognosis factors for ICU and one-year mortality. Multivariable logistic regression results adjusted for gender and age at ICU admission. ADE: acquired immunodeficiency syndrome (AIDS)-defining events; ART: antiretroviral therapy; ICU: intensive care unit; IMV: invasive mechanical ventilation; RRT: renal replacement therapy; SOFA: Sepsis-related Organ Failure Assessment. Definitions can be found at electronic supplementary material 1

ADE-related admissions (OR 2.38 [1.09–5.22]; p = 0.030), ICU complications (OR 2.28 [1.04–4.96]; p = 0.038), higher SOFA scores at admission (OR 1.11 [1.01–1.23]; p = 0.037), IMV (OR 7.51 [2.46–22.95]; p < 0.001) and RRT requirement (OR 3.79 [1.26–11.44]; p = 0.018) predicted ICU mortality, whereas ART use during ICU admission (OR 0.42 [0.21–0.88]; p = 0.021) was protective. For one-year mortality, higher SOFA scores at admission (OR 1.13 [1.05–1.22]; p = 0.002), admission from hospital wards (OR 2.97 [1.54–5.72]; p = 0.005) and more comorbidities (OR 1.52 [1.20–1.91]; p < 0.001), were significant predictors (Fig. 3). ST4 and ST5 show univariable analysis for ICU and one-year mortality.

## Discussion

This study confirms a significant decrease in mortality among critically ill PLHIV when comparing the periods 2006–2015 and 2016–2019. The reduction in mortality is attributable to changes in patients' characteristics.

Despite advancements in ART access and management, especially in Western and Central Europe and North America [1, 2], the primary characteristics of PLHIV have remained relatively consistent over the study periods. Most admissions (82%) were previously on-ART; however, despite a non-significant trend towards an increase in pre-admission CD4 count (250 vs 287.5 cells/mm3, OR 1.02 [0.98–1.06]), nearly 40% of these patients had detectable VL and advanced HIV infection (CD4 cell count < 200 cells/mm^3^), with ART typically initiated approximately two years after diagnosis.

A shift in comorbidity patterns over time has been observed. The prevalence of IVDU and related comorbidities, such as HCV co-infection and liver cirrhosis, has decreased. In contrast, comorbidities associated with aging and cardiovascular risk have increased, consistent with findings from other studies [7, 15–17]. This trend reflects changes in drug consumption patterns within the population [2], improved infection control, and increased life expectancy. Consequently, there are now more opportunities for developing comorbidities related to age, lifestyle, and ART [8, 18, 19].

Infectious events, especially respiratory infections, were the main causes of ICU admissions, particularly in the earlier period, in line with recent literature [20]. ADEs accounted for 22% of admissions, a rate consistent with other studies, though we did not observe a decrease in ADE-related admissions in recent years reported by others [4, 6, 7]. This may be due to stable rates of late HIV diagnoses within our ICU admissions, that account for most of ADEs admissions, and it is lower than recent published data by others [2, 7].

Supportive measures such as IMV and vasopressor use have decreased over time in our cohort. This differs from other studies showing stable IMV rates but decreased vasopressor use [4, 6, 7]. Similarly, our cohort had lower rates of RRT compared to others [4, 6, 7]. Differences in admission policies, critical patient management and the period of analysis may potentially justify these differences.

A notable reduction in ICU complications was observed, likely due to improved critical care management and preventive measures against ICU-acquired infections. Spanish National Safety Program, initiated in 2009, has contributed to significant reductions in infection rates, aligning with our findings [21].

We observed a significant increase in ART use at ICU discharge (72% vs. 85%) and a non-significant increase at hospital discharge (87% vs. 92%) and 12-monts follow up (87% vs 90%) between the first and second period, respectively. This change aligns with the main clinical guidelines for HIV treatment, which since 2016 recommend ART initiation regardless of immune status [22–25].

A significant decrease in mortality, particularly in the ICU, with a trend toward reduced mortality at one-year post-discharge was found. Multivariable adjustment showed that this reduction was associated with differences in patient characteristics and management between the two periods. As previously noted, the need for IMV, a critical factor associated with mortality, was significantly less prevalent in the second period, as were ICU complications. Notably, the decrease in IMV requirements in the most recent period is a novel finding not yet described in the literature.

Large studies have demonstrated significant improvements in survival among PLHIV due to widespread ART use and effective immunovirological control [26–29]. However, few studies have evaluated recent changes in ICU mortality among PLHIV [4, 6, 7, 9, 15, 30–32]. Typically, ICU mortality has been associated with severity at admission [4, 6, 7, 9, 15, 30], while later mortality has been attributed to virological control and immunological status [31, 32]. Our data support some of these previous findings, showing that severity-associated factors such as the need for IMV, RRT, SOFA score and ICU complications influence ICU mortality.

We found that ART use during ICU was a protective factor against ICU mortality. This finding should be interpreted with caution. Although 82% of our entire cohort was on ART before becoming critically ill, ART use during ICU admission dropped to 53%. This reduction reflects the challenges in maintaining oral treatment, managing side effects, dealing with impaired renal and hepatic function, and navigating drug-drug interactions [33, 34]. Consequently, the decision to maintain or initiate ART during ICU admission depends on several factors, which can result in differences between patient groups. We did not assess the exact timing of treatment suspension, the specific reasons for this reduction, or the characteristics of patients who continued or did not continue on ART at ICU admission. However, the proportion of patients using ART in the ICU was similar across both periods (50% in the first period and 59% in the second), particularly for those who were ART-naïve (7% vs. 6%). Although there is no clear data supporting early ART initiation in ICU [35], it is generally recommended to avoid ART discontinuation for more than two weeks to prevent resistance and future HIV treatment failure [33]. Significant changes between periods were observed in the type of ART regimen used, with a decrease in non-nucleoside reverse transcriptase inhibitors (NNRTIs) and boosted protease inhibitors (PIs) in the second period, and a shift towards integrase inhibitors (INSTIs)-based regimens. The safer profile of the latter in terms of drug-drug-interactions, tolerability, and use in patients with impaired organ function, will likely contribute to increase the use of ART in the ICU and improve outcomes. A multidisciplinary approach is essential to further increase the rate of ART use during ICU admission.

We did not find virological and immunological status variables associated with one-year mortality among ICU survivors. In contrast, we found that characteristics of ICU admission (SOFA score) and being admitted from the ward (compared to being admitted from the emergency department), may also influence one-year prognosis, together with the number of comorbidities. These results align with other studies, although most do not have a one-year follow-up [7, 17, 19, 26, 36, 37]. Additionally, numerous analyses in critically ill patients (not specifically PLHIV) have shown that ICU admission is associated with increased medium-to-long-term mortality, primarily linked to the Post-ICU Syndrome and increased vascular risk [38, 39]. The lack of influence of immunovirological variables on medium-term survival could be related to the high proportion of patients on ART at ICU discharge. Regarding this point, while most studies in the field have presented data on ART use before ICU admission and some during ICU stay, there is a lack of information on ART use upon ICU discharge. Failure to continue ART after ICU and hospitalization may be one of the main modifiable factors associated with long-term mortality, making it essential to investigate about the elements that influence this situation.

This study has limitations, including its retrospective, observational, and single-center design, which may affect the generalizability of the results. However, it provides valuable insights into the impact of universal ART recommendations on critically ill PLHIV, incorporating extensive data on admission characteristics, immunological status, and follow-up outcomes up to 12 months post-discharge.

## Conclusions

In conclusion, ICU and one-year mortality among PLHIV requiring ICU admission have decreased since the extension of ART to all patients, regardless of immunological status, in 2016. This reduction is primarily due to changes in patient profiles, including lower severity and support needs at admission. Further studies are needed to confirm these results and explore factors influencing long-term outcomes.

## Supplementary Information

Below is the link to the electronic supplementary material.

Supplementary file1 (PDF 812 KB)

## Data Availability

The data supporting the findings of this study are available from the corresponding author upon reasonable request.
