# Supplementary material for: Epidemiological changes and outcomes of people living with HIV admitted to the intensive care unit: a 14-year retrospective study
Source: Infection. 2024 Oct 11;53(2):583–92. doi: 10.1007/s15010-024-02402-x (PMC11971213; doi:10.1007/s15010-024-02402-x)
Supplement: Supplementary file 1 — Supplementary file1 (PDF 812 KB) [file 15010_2024_2402_MOESM1_ESM.pdf]

SUPPLEMENTARY INFORMATION (SI)

Article title: Epidemiological Changes and Outcomes of People Living with HIV Admitted to the Intensive Care Unit: A 14-Year Retrospective Study.

Journal Name: Infection

Author names: Esther Martínez RN, MSN[1,2,3], Alberto Foncillas MD[4], Adrián Téllez MD, PhD[5], Sara Fernández MD[5], Gemma Martínez-Nadal MD[5,6], Verónica Rico MD[4,5,7], Adrià Tomé MD[5,8], Ainoa Ugarte MD, PhD[4,5,7], Mariano Rinaudo MD, PhD[5,9], Leire Berrocal MSc[4,10], Elisa De Lazzari MSc[3,4,10,11], Jose M Miró MD, PhD[3,4,10,11], Jose M Nicolás MD, PhD[3,5,,10], Josep Mallolas MD, PhD[3,4,10,11], Lorena De la Mora MD, PhD[4,5], Pedro Castro MD, PhD[3,5,10,11]

Affiliation:

[1] Intensive Care Unit, Hospital General de Granollers, Barcelona, España

[2] Intensive Care Unit, Hospital Sant Joan Despí Moisès Broggi, Barcelona, Spain

[3] University of Barcelona, Spain

[4] Infectious Diseases Department, Hospital Clínic, Barcelona, España

[5] Medical Intensive Care Unit, Hospital Clínic, Barcelona, Spain

[6] Emergency Department, Hospital Clínic, Barcelona, Spain

[7] Hospital at Home, Hospital Clínic, Barcelona, Spain

[8] Emergency Department, Complejo Hospitalario Universitario A Coruña, Spain

[9] Intensive Care Unit, Hospital Universitari de Vic, Spain

[10] IDIBAPS, Barcelona, Spain

[11] CIBERINFEC, Instituto de Salud Carlos III, Madrid, Spain

E-mail address of the corresponding author: Pedro Castro, Medical Intensive Care Unit, Hospital Clínic, Barcelona, 170 Villarroel St, 08030, Barcelona, Spain. pcastro@clinic.cat

- **SI1: Definition and measurement of variables collected (by alphabetical order)**
- **Supplemental Table 1 (ST1): General, HIV infection-related, and ICU admission / evolution characteristics of admissions included in the study (total and by periods, univariable analysis)**
- **Supplemental Table 2 (ST2): General, HIV infection-related, and ICU admission / evolution characteristics of the ICU survivors included in the study (total and by periods, univariable analysis)**
- **Supplemental Table 3 (ST3): Differences among periods (2006-2015 vs 2016-2019). Multivariable logistic regression results adjusted for gender and age at ICU admission.**
- **Supplemental Table 4 (ST4): Predictors of ICU mortality. Characteristics related to ICU outcomes of the patients included in the study (comparison between survivors and deceased). Univariable analysis.**
- **Supplemental Table 5 (ST5): Predictors of one-year mortality among ICU survivors. Characteristics related to ICU discharge outcomes of the patients included in the study**

that survived ICU admission (comparison between survivors and deceased). Univariable analysis.

SI1: Definition and measurement of variables collected (by alphabetical order)

- **Acute Physiology And Chronic Health Evaluation (APACHE) II score:** A score used to measure disease severity for adult patients admitted to the Intensive Care Unit (ICU) (1). It is based on 12 current physiological measurements, age, and previous health conditions. An increasing score (range 0 to 71) correlates with the subsequent risk of hospital death. It was performed within 24 hours of ICU admission.
- **Acquired Immunodeficiency Syndrome (AIDS)-defining event (ADE):** Any HIV-related illness included in the Centers for Disease Control and Prevention's (CDC) list of diagnostic criteria for AIDS (2). ADEs include opportunistic infections and cancers that are life-threatening in a person living with HIV: esophageal, tracheal, bronchial or pulmonary candidiasis, invasive cervical cancer, coccidioidomycosis, cryptococcosis, cryptosporidiosis, cystoisosporiasis, cytomegalovirus, HIV-related encephalopathy, herpes simplex virus, histoplasmosis, Kaposi's sarcoma, lymphoma, extrapulmonary tuberculosis, mycobacterium avium complex, pneumocystis pneumonia, recurrent pneumonia, progressive multifocal leukoencephalopathy, salmonella septicemia, toxoplasmosis, and wasting syndrome due to HIV.
- **Non-AIDS-defining event (non-ADE):** Any HIV-related illness not included in the Centers for Disease Control and Prevention's (CDC) list of diagnostic criteria for AIDS. Non-ADEs include infections (non-opportunistic), cancer, and cardiovascular, liver, kidney, bone, and neuropsychiatric diseases.
- **Antiretroviral treatment (ART) adherence:** The act of taking a treatment exactly as prescribed. This was obtained from medical reports.
- **ART regimens:** Combination of antiretroviral therapy.
- **Cardiopathy (comorbidities):** Includes cardiopathy of any origin, including ischemic heart disease, or heart failure.
- **Cardiovascular process:** Includes myocardial infarction, arrhythmias, any kind of shock and any other cardiovascular process causing ICU admission.
- **Catheter-related bloodstream infection (CRBSI):** Defined as the presence of bacteremia originating from an intravenous catheter.
- **CD4 lymphocyte count in ICU:** The number of CD4 T cells measured during the stay in ICU. If measured more than once, the first value was collected.
- **CD4 lymphocyte count nadir or Nadir CD4+:** The lowest ever measured CD4 T cell count before ICU admission.
- **CD4 lymphocyte count pre-admission:** The last CD4 T cell count measured before ICU admission.
- **Comorbidities:** Include the following conditions: cardiopathy, hypertension, dyslipidemia, diabetes mellitus, chronic kidney disease, Chronic Obstructive Pulmonary Disease (COPD), neoplasm, stroke, chemotherapy (current or within the last 5 years), active immunosuppressive therapy, splenectomy, immunosuppressive-dose corticosteroids, home non-invasive mechanical ventilation (NIMV), home oxygen, severe pulmonary hypertension o under treatment.
- **Complications in ICU:** Include the following situations occurring during ICU admission: CRBSI, ventilator-associated pneumonia (VAP), urinary tract infection (UTI), pneumothorax, systemic candidiasis, skin infection, central nervous system hemorrhage, gastrointestinal hemorrhage, deep venous thrombosis, pulmonary embolism, *de novo* atrial fibrillation, cardiorespiratory arrest, cytomegalovirus reactivation, acute myocardial infarction.

- **Digestive process:** Includes upper or lower gastrointestinal bleeding, pancreatitis, surgical abdominal illness, or other abdominal processes causing ICU admission.
- **Discontinued ART at ICU discharge:** Patients that do not continue or do not start ART after ICU discharge (regardless of the reason)**.**
- **First CD4 count post-ICU discharge:** The first count of CD4 T lymphocytes after ICU discharge.
- **Hepatic process:** Includes hepatic encephalopathy, acute liver failure and any other liver diseases causing ICU admission.
- **ICU readmissions during the same hospitalization:** When a patient is discharged from ICU and readmitted to the ICU before being discharged from the hospital.
- **Infectious process:** Includes any of the following diagnosis (either community or hospital-acquired): meningoencephalitis, pneumonia and other respiratory infections, abdominal infection, cholangitis, cholecystitis, endocarditis, cutaneous infection, bacteremia, urinary tract infection, and any other infection.
- **Invasive mechanical ventilation (IMV) during ICU admission:** Respiratory support with positive pressure to the lungs via an artificial airway (tracheal intubation or tracheostomy).
- **Late Human Immunodeficiency Virus (HIV) diagnosis:** When the diagnosis of HIV infection is performed once the patient presents a CD4 T cell count < 350 cells/µL or with an ADE regardless of the CD4 T cell count.
- **Neoplasms (comorbidities):** Active solid or hematological neoplasia under treatment or diagnosed within the last 5 years, excluding non-melanoma skin cancer.
- **Neurological process:** Includes epileptic crisis and *status epilepticus*, subarachnoid hemorrhage, coma, delirium or agitation, traumatic brain injury, mass effect in the central nervous system, stroke, and other neurological processes causing ICU admission.
- **Opportunistic Infection (OI):** An infection that occurs more frequently or is more severe in people with weakened immune systems, such as those with low CD4 T cell counts. They include any of the following: esophageal, tracheal, bronchial or pulmonary candidiasis, coccidioidomycosis, cryptococcosis, cryptosporidiosis, cystoisosporiasis, cytomegalovirus, HIV-related encephalopathy, herpes simplex virus, histoplasmosis, extrapulmonary tuberculosis, mycobacterium avium complex, pneumocystis pneumonia, recurrent pneumonia, progressive multifocal leukoencephalopathy, salmonella septicemia, toxoplasmosis.
- **Post-ICU Syndrome:** A collection of physical, cognitive, and mental health symptoms that some individuals experience after being discharged from an ICU.
- **Pre-admission viral load (VL):** Last known VL measurement before ICU admission.
- **Renal replacement therapy (RRT):** Includes both continuous and intermittent renal replacement therapy.
- **Respiratory infection**: Includes pneumonia (including that caused by *Pneumocystis jirovecii*) or non-pneumonic respiratory infection.
- **Respiratory process:** Includes pulmonary embolism, pneumothorax, decompensated asthma, decompensated COPD, and other pulmonary diseases causing ICU admission.
- **Sequential Organ Failure Assessment (SOFA):** A score designed to describe organ dysfunction/failure in critically ill patients. The score is based on dysfunction of six different organs or systems: respiratory, cardiovascular, hepatic, coagulation, renal, and neurological. Scores ranges from 0 (best) to 24 (worst) points (3). It was measured upon admission and at 48 hours.
- **Undetectable VL:** A level of VL that is too low to be measured by the VL test being used or below an agreed threshold. In our cohort a value of 50 copies/ml was used as a threshold.

- **Vasopressors**: Include norepinephrine, epinephrine or/and dobutamine.
- **VL in ICU or ICU VL:** VL measured during ICU stay. If measured more than once, the first value was collected.

**ST1. General, HIV infection-related, and ICU admission / evolution characteristics of admissions included in the study (total and by periods, univariable analysis)**					
**Variables**[a]	**Total (n=502)**	**2006-2015 (n=328)**	**2016-2019 (n=174)**	**OR (95% CI)**	**p Value**
**General characteristics**					
Sex (male)[b]	375 (75%)	249 (76%)	126 (72%)	-*	-*
Age (years)[b]	47.5 (39.7-53.9)	46.1 (39.6-52.4)	50 (41.5-57.3)	-*	-*
Toxic habits					
Tobacco smoking	355 (71%)	238 (73%)	117 (67%)	0.78 (0.50-1.20)	0.256
IVDU[c]	179 (36%)	139 (42%)	40 (23%)	0.40 (0.24-0.67)	**0.001**
Alcohol	139 (28%)	102 (31%)	37 (21%)	0.60 (0.37-0.96)	**0.035**
Comorbidities					
Number of comorbidities	0 (0-2)	0 (0-1)	1 (0-2)	1.09 (0.91-1.31)	0.336
Hypertension	72 (14%)	41 (12%)	31 (18%)	1.26 (0.69-2.28)	0.455
Dyslipidaemia	59 (12%)	29 (9%)	30 (17%)	1.86 (1.00-3.47)	0.052
Diabetes mellitus	48 (10%)	29 (9%)	19 (11%)	1.05 (0.52-2.09)	0.895
Cardiopathy	46 (9%)	32 (10%)	14 (8%)	0.69 (0.32-1.47)	0.335
Chronic kidney disease	27 (5%)	15 (5%)	12 (7%)	1.49 (0.59-3.78)	0.400
COPD (GOLD IV)	19 (4%)	9 (3%)	10 (6%)	1.84 (0.35-9.64)	0.473
Neoplasms	75 (15%)	47 (14%)	28 (16%)	1.03 (0.59-1.81)	0.915
Co-infections by hepatotropic viruses					
HBV	82 (16%)	57 (17%)	25 (14%)	0.78 (0.44-1.39)	0.397
HCV	226 (45%)	161 (49%)	65 (37%)	0.61 (0.40-0.93)	**0.022**
Liver cirrhosis	116 (23%)	85 (26%)	31 (18%)	0.59 (0.34-1.02)	0.058
Decompensated	67 (58%)	47 (55%)	20 (65%)	1.34 (0.53-3.35)	0.535
**HIV infection-related characteristics**					
Previous HIV diagnosis at admission	459 (91%)	299 (91%)	160 (92%)	0.96 (0.48-1.90)	0.902
Time between HIV diagnosis and admission to hospital (years) (n=420)	15.71 (7.41-21.68)	13.41 (6.31-19.31)	20.17 (9.77-25.72)	1.08 (1.04-1.11)	**<0.001**
Undetectable VL[d] pre-admission (n=403)	257 (64%)	156 (60%)	101 (70%)	1.34 (0.85-2.12)	0.205
Ever had undetectable VL[d] (n=388)	335 (86%)	204 (83%)	131 (93%)	2.45 (1.17-5.14)	**0.018**
CD4+ count pre-admission (cells/mm^3) (n=398)	255 (102-473)	250 (120-450)	287.5 (81-536)	1.02 (0.98-1.06)[e]	0.268
CD4+ count <200 cells/mm^3 pre-admission (n=398)	157 (39%)	103 (40%)	54 (39%)	1.01 (0.63-1.62)	0.976
Nadir CD4+ (cells/mm^3) (n=400)	88 (27.5-204.5)	94 (32-210)	82 (24-185)	0.98 (0.92-1.05)[e]	0.611
Months between nadir CD4+ count and admission (n=397)	19.98 (1.31-89.07)	11.07 (0.89-60.44)	51.32 (4.53-164.27)	1.01 (1.00-1.01)	**<0.001**
Years between diagnosis and first ART (n=277)	2.34 (0.28-10.97)	2.82 (0.27-11.75)	2.00 (0.29-9.04)	0.98 (0.94-1.03)	0.418
ART pre-admission (n=445)	367 (82%)	230 (79%)	137 (88%)	1.73 (0.92-3.23)	0.087

ART family used pre-admission (n=356)					
INSTIs	85 (24%)	19 (9%)	66 (50%)	1	**<0.001**
NNRTIs	81 (23%)	58 (26%)	23 (17%)	0.11 (0.05-0.24)	
PIs	106 (30%)	87 (39%)	19 (14%)	0.06 (0.03-0.14)	
Other ART groups	84 (24%)	59 (26%)	25 (19%)	0.12 (0.05-0.28)	
Proper adherence to ART in the last 6 months (n=278)	220 (79%)	132 (78%)	88 (81%)	1.15 (0.56-2.34)	0.705
Months on ART pre-admission (n=265)	15.17 (4.53-28.68)	13.79 (4.10-27.86)	16.88 (5.15-30.91)	1.00 (0.99-1.01)	0.426
Period on ART (n=296)					
Less than 3 months	25 (8%)	19 (10%)	6 (5%)	1	**0.043**
Between 3-12 monts	22 (7%)	19 (10%)	3 (3%)	0.51 (0.10-2.68)	
More than 12 months	249 (84%)	145 (79%)	104 (92%)	2.05 (0.69-6.10)	
Pre-admission OI prophylaxis (n=417)	86 (21%)	62 (23%)	24 (17%)	0.71 (0.42-1.20)	0.202
**ICU admission / evolution characteristics**					
Other causes of ICU admission[f]					
Cardiovascular process	146 (29%)	100 (30%)	46 (26%)	0.79 (0.52-1.21)	0.283
Respiratory process	87 (17%)	52 (16%)	35 (20%)	1.35 (0.73-2.50)	0.336
Hepatic process	32 (6%)	22 (7%)	10 (6%)	0.79 (0.33-1.89)	0.603
Digestive process	55 (11%)	37 (11%)	18 (10%)	0.90 (0.49-1.67)	0.739
Neurological process	80 (16%)	47 (14%)	33 (19%)	1.47 (0.88-2.47)	0.141
Surgical process (elective and non-elective)	121 (24%)	87 (27%)	34 (20%)	0.64 (0.40-1.03)	0.141
Oncological or hematological process	41 (8%)	24 (7%)	17 (10%)	1.31 (0.60-2.85)	0.499
Intoxication or adverse reaction	36 (7%)	25 (8%)	11 (6%)	0.99 (0.46-2.11)	0.971
Need of IMV	216 (43%)	170 (52%)	46 (26%)	0.34 (0.22-0.51)	**<0.001**
Duration of IMV (days) (n=206)	4 (1-9)	4 (1-9)	5 (1-7)	0.99 (0.97-1.02)	0.664
Need of NIV	68 (13%)	46 (14%)	22 (13%)	0.83 (0.43-1.62)	0.583
Duration of NIV (days) (n=62)	2 (1-3)	2 (1-3)	2 (1.5-3)	0.95 (0.87-1.04)	0.286
Undetectable VL[d] during admission (n=247)	112 (45%)	79 (46%)	33 (44%)	0.82 (0.46-1.48)	0.515
ART during ICU admission (n=429)	228 (53%)	140 (50%)	88 (59%)	1.40 (0.89-2.20)	0.144
ART family used during ICU admission (n=226)					
INSTIs	61 (27%)	17 (12%)	44 (51%)	1	**<0.001**
NNRTIs	49 (22%)	37 (26%)	12 (14%)	0.12 (0.04-0.31)	
PIs	67 (30%)	54 (39%)	13 (15%)	0.10 (0.04-0.25)	
Other ART groups	49 (22%)	32 (23%)	17 (20%)	0.18 (0.07-0.48)	
Days from ICU discharge to hospital discharge (n=500)	7 (2-17)	7 (2-18)	6 (2-16)	1.00 (0.99-1.01)	0.652

[a]Under each variable, the sample size (n) on which the analysis was conducted is provided if it is not the whole cohort (patients for whom the information was not available are excluded). [b]Used as adjustment variable; [c]Past and current; [d]<50 copies/mL; [e]Per increase of 50 cells/mm^3; [f]One patient could share several categories. However, infections are excluded from cardiovascular, surgical, respiratory, and neurological processes * These variables are for adjustment, and their odds ratio (OR) is not interpreted.

Categorical variables are expressed as n (%) and quantitative variables as median (P25-P75). ART: antiretroviral therapy; 95% CI: 95% confidence interval; COPD: chronic obstructive pulmonary disease; HBV: hepatitis B virus; HCV: hepatitis C virus; HIV: human immunodeficiency virus; ICU: intensive care unit; IMV: invasive mechanical ventilation; INSTIs: integrase inhibitors; IVDU: intravenous drug users; NIV: non-invasive ventilation; NNRTIs: non-nucleoside reverse transcriptase inhibitors; OI: opportunistic infection; OR: odds ratio; PIs: protease inhibitors; VL: viral load (copies/mL).

ST2. General, HIV infection-related, and ICU admission / evolution characteristics of the ICU survivors included in the study (total and by periods, univariable analysis)					
**Variables**[a]	**Total (n=443)**	**2006-2015 (n=281)**	**2016-2019 (n=162)**	**OR (95% CI)**	**p Value**
**General characteristics**					
Sex (male)[b]	326 (74%)	210 (75%)	116 (72%)	-*	-*
Age (years)[b]	47 (39.7-53.9)	45.9 (39.6-51.8)	50.1 (42.2-57.4)	-*	-*
Toxic habits					
Tobacco smoking	310 (70%)	202 (72%)	108 (67%)	0.78 (0.49-1.24)	0.302
IVDU[c]	158 (36%)	121 (43%)	37 (23%)	0.39 (0.23-0.67)	**0.001**
Alcohol	121 (27%)	85 (30%)	36 (22%)	0.67 (0.41-1.10)	0.110
Comorbidities					
Number of comorbidities	0 (0-2)	0 (0-1)	1 (0-2)	1.05 (0.86-1.27)	0.648
Hypertension	67 (15%)	37 (13%)	30 (19%)	1.21 (0.65-2.25)	0.555
Dyslipidemia	54 (12%)	27 (10%)	27 (17%)	1.55 (0.79-3.02)	0.199
Diabetes mellitus	46 (10%)	27 (10%)	19 (12%)	1.00 (0.49-2.05)	0.992
Cardiopathy	43 (10%)	30 (11%)	13 (8%)	0.60 (0.27-1.31)	0.198
Chronic kidney disease	24 (5%)	12 (4%)	12 (7%)	1.75 (0.67-4.55)	0.250
COPD (GOLD IV)	17 (4%)	7 (2%)	10 (6%)	2.16 (0.38-12.17)	0.381
Neoplasms	64 (14%)	42 (15%)	22 (14%)	0.80 (0.43-1.48)	0.473
Co-infections by hepatotropic viruses					
HBV	77 (17%)	52 (19%)	25 (15%)	0.79 (0.44-1.42)	0.426
HCV	196 (44%)	138 (49%)	58 (36%)	0.57 (0.36-0.89)	**0.014**
Liver cirrhosis	98 (22%)	72 (26%)	26 (16%)	0.53 (0.30-0.93)	**0.028**
Decompensated	57 (58%)	40 (56%)	17 (65%)	1.26 (0.46-3.45)	0.655
**HIV infection-related characteristics**					
Previous HIV diagnosis at admission	405 (91%)	256 (91%)	149 (92%)	0.98 (0.48-2.02)	0.965
Time between HIV diagnosis and admission to hospital (years) (n=371)	15.72 (7.39-21.56)	13.48 (6.32-18.74)	20.17 (9.65-25.72)	1.07 (1.04-1.11)	**<0.001**
Undetectable VL[d] pre-admission (n=355)	234 (66%)	137 (62%)	97 (72%)	1.28 (0.77-2.11)	0.341
Ever had undetectable VL[d] (n=338)	295 (87%)	172 (83%)	123 (94%)	2.67 (1.17-6.09)	**0.019**
CD4+ count pre-admission (cells/mm^3) (n=350)	290 (121-493)	275 (127-458)	290 (90-543)	1.02 (0.98-1.06)[e]	0.458
CD4+ count <200 cells/mm^3 pre-admission (n=350)	130 (37%)	81 (37%)	49 (37%)	1.07 (0.65-1.77)	0.789
Nadir CD4+ (cells/mm^3) (n=345)	93 (30-205)	101 (33-210)	82 (25-189)	0.99 (0.91-1.07)[e]	0.717
Months between nadir CD4+ count and admission (n=343)	21.98 (1.35-91.01)	11.84 (0.89-60.44)	52.21 (4.37-164.27)	1.01 (1.00-1.01)	**<0.001**
Years between diagnosis and first ART (n=244)	2.07 (0.25-10.76)	2.17 (0.24-10.99)	1.98 (0.32-9.59)	0.99 (0.95-1.04)	0.692
ART pre-admission (n=394)	326 (83%)	196 (79%)	130 (89%)	1.83 (0.93-3.58)	0.079

Proper adherence to ART in the last 6 months (n=247)	198 (80%)	114 (79%)	84 (82%)	1.03 (0.49-2.15)	0.935
Months on ART pre-admission (n=233)	15.27 (4.76-29.53)	13.60 (4.40-27.86)	17.31 (4.92-31.24)	1.00 (0.99-1.01)	0.445
More than 12 months on ART (n=263)	224 (85%)	124 (80%)	100 (93%)	1.80 (0.59-5.51)	**0.050**
Pre-admission OI prophylaxis (n=369)	71 (19%)	49 (21%)	22 (16%)	0.76 (0.43-1.35)	0.347
**ICU admission / evolution characteristics**					
Admission source					
Emergency department	265 (60%)	165 (59%)	100 (62%)	1	0.591
Hospital ward	92 (21%)	59 (21%)	33 (20%)	0.95 (0.56-1.61)	
Others (operating room, another ICU)	86 (19%)	57 (20%)	29 (18%)	0.76 (0.44-1.29)	
Days of hospitalization before ICU[f] (n=92)	8 (3.5-16)	8 (4-19)	6 (3-14)	0.98 (0.94-1.03)	0.398
Type of admission (medical/surgical)					
Medical admission	333 (75%)	204 (73%)	129 (80%)	1	0.092
Surgical admission (elective)	74 (17%)	48 (17%)	26 (16%)	0.76 (0.44-1.32)	
Surgical admission (urgent)	36 (8%)	29 (10%)	7 (4%)	0.41 (0.17-0.96)	
Main cause of ICU admission[g]					
Infectious process	227 (51%)	145 (52%)	82 (51%)	1.05 (0.70-1.56)	0.814
Respiratory infection	134 (30%)	97 (35%)	37 (23%)	0.60 (0.38-0.95)	**0.031**
ADE	89 (20%)	57 (20%)	32 (20%)	1.05 (0.64-1.74)	0.840
OI	78 (18%)	51 (18%)	27 (17%)	1.02 (0.61-1.72)	0.926
Cardiovascular process	116 (26%)	77 (27%)	39 (24%)	0.79 (0.50-1.26)	0.329
Respiratory process	81 (18%)	47 (17%)	34 (21%)	1.39 (0.74-2.61)	0.313
Hepatic process	26 (6%)	18 (6%)	8 (5%)	0.69 (0.26-1.80)	0.447
Digestive process	44 (10%)	29 (10%)	15 (9%)	0.86 (0.43-1.74)	0.680
Neurological process	69 (16%)	39 (14%)	30 (19%)	1.50 (0.87-2.59)	0.148
Surgical process (elective and urgent)	114 (26%)	80 (28%)	34 (21%)	0.63 (0.39-1.02)	0.062
Oncological or hematological process	32 (7%)	19 (7%)	13 (8%)	1.10 (0.43-2.81)	0.840
Intoxication or adverse reaction	34 (8%)	23 (8%)	11 (7%)	0.99 (0.46-2.16)	0.990
APACHE II (admission)	18 (14-21)	18 (14-21)	18 (13-21)	0.98 (0.94-1.02)	0.284
SOFA (admission)	5 (3-8)	6 (3-9)	4 (2-7)	0.91 (0.85-0.97)	**0.005**
SOFA at 48h	4 (2-6)	4 (3-6)	4 (2-5)	0.91 (0.84-0.98)	**0.017**
Need of IMV	164 (37%)	126 (45%)	38 (23%)	0.38 (0.24-0.59)	**<0.001**
Duration of IMV (days) (n=155)	4 (1-7)	4 (1-8)	3 (1-7)	0.97 (0.92-1.02)	0.171
Need of NIV	56 (13%)	36 (13%)	20 (12%)	0.91 (0.45-1.86)	0.797
Duration of NIV (days) (n=51)	2 (1-3)	2 (1-3)	2 (2-3)	0.95 (0.86-1.04)	0.275
Vasopressors	172 (39%)	125 (44%)	47 (29%)	0.51 (0.33-0.78)	**0.002**

Norepinephrine	170 (38%)	124 (44%)	46 (28%)	0.50 (0.32-0.78)	**0.002**
Epinephrine	11 (2%)	7 (2%)	4 (2%)	0.91 (0.27-3.04)	0.876
Dobutamine	23 (5%)	14 (5%)	9 (6%)	0.97 (0.39-2.45)	0.954
RRT	22 (5%)	9 (3%)	13 (8%)	2.88 (1.06-7.79)	**0.037**
Total parenteral nutrition	66 (15%)	51 (18%)	15 (9%)	0.46 (0.24-0.87)	**0.017**
ART during ICU admission (n=379)	210 (55%)	126 (52%)	84 (61%)	1.38 (0.86-2.22)	0.183
ART family used during ICU admission (n=208)					
INSTIs	60 (29%)	16 (13%)	44 (54%)	1	**<0.001**
NNRTIs	46 (22%)	34 (27%)	12 (15%)	0.12 (0.04-0.32)	
PIs	59 (28%)	47 (37%)	12 (15%)	0.10 (0.04-0.25)	
Other ART groups	43 (21%)	29 (23%)	14 (17%)	0.16 (0.06-0.43)	
VL (copies/mL) in ICU (n=215)	101 (49-105900)	119.5 (49-111900)	101 (49-82300)	1 (1-1)	0.390
Undetectable VL[d] during admission (n=215)	98 (46%)	66 (46%)	32 (45%)	0.86 (0.46-1.62)	0.646
CD4+ count (cells/mm^3) in ICU (n=201)	114 (37-257)	116 (41-249)	80 (29-272)	0.98 (0.91-1.05)[e]	0.490
Complications in ICU (n=441)	169 (38%)	126 (45%)	43 (27%)	0.46 (0.30-0.70)	**<0.001**
Surgical wound infection	38 (9%)	36 (13%)	2 (1%)	0.08 (0.02-0.36)	**0.001**
UTI	22 (5%)	15 (5%)	7 (4%)	0.70 (0.27-1.84)	0.466
VAP	18 (4%)	15 (5%)	3 (2%)	0.31 (0.09-1.10)	0.070
CRBSI	19 (4%)	16 (6%)	3 (2%)	0.29 (0.08-1.08)	0.064
Non-CRBSI	33 (7%)	30 (11%)	3 (2%)	0.16 (0.05-0.55)	**0.004**
ICU length of stay (days)	4 (2-8)	4 (2-8)	4 (2-7)	0.99 (0.97-1.01)	0.399
Hospital length of stay (days) (n=441)	17 (9-34)	18 (9-35.5)	16 (8-29)	1.00 (0.99-1.01)	0.528
Days from ICU discharge to hospital discharge (n=441)	8 (4-19)	9 (4-20)	7 (4-17)	1.00 (0.99-1.01)	0.895
ICU readmission during the same stay (n=324)	25 (8%)	10 (5%)	15 (11%)	2.12 (0.91-4.94)	0.083

[a]Under each variable, the sample size (n) on which the analysis was conducted is provided if it is not the whole cohort (patients for whom the information was not available are excluded); [b]Used as adjustment variable; [c]Past and current; [d]<50copies/mL; [e]Per increase of 50 cells/mm$^{3;}$ [f]Only for patients admitted from wards; patients coming from emergency department are excluded; [g]One patient could share several categories. However, infections are excluded from cardiovascular, surgical, respiratory, and neurological processes. * These variables are for adjustment, and their odds ratio (OR) is not interpreted.

Categorical variables are expressed as n (%) and quantitative variables as median (P25-P75). ADE: acquired immunodeficiency syndrome (AIDS)-defining event; APACHE II: Acute Physiology and Chronic Health Evaluation; ART: antiretroviral therapy; CI: confidence interval; COPD: chronic obstructive pulmonary disease; CRBSI: catheter related bloodstream infection; HBV: hepatitis B virus; HCV: hepatitis C virus; HIV: human immunodeficiency virus; ICU: intensive care unit; IMV: invasive mechanical ventilation; INSTIs: integrase inhibitors; IVDU: intravenous drug users; NIV: non-invasive ventilation; NNRTIs: non-nucleoside reverse transcriptase inhibitors; OI: opportunistic infection; OR: odds ratio; PIs: protease inhibitors; RRT: renal replacement therapy; SOFA: Sepsis-related Organ Failure Assessment; UTI: urinary tract infection; VAP: ventilator-associated pneumonia; VL: viral load (copies/mL).

ST3. Differences among periods (2006-2015 vs 2016-2019). Multivariable logistic regression results adjusted for gender and age at ICU admission			
**Reference group: Period 2006-2015 (total of admissions) (n=444)**		**OR (95% C.I.)**	**p Value**
	IVDU	0.41 (0.24-0.72)	0.002
	Non-CRBSI	0.13 (0.03-0.56)	0.006
	IMV	0.42 (0.26-0.68)	<0.001
	Surgical wound infection	0.04 (0.00-0.28)	0.001
	Main cause of ICU admission: surgical process	0.55 (0.32-0.95)	0.033
	Pre-admission ART	2.03 (1.00-4.11)	0.049
**Reference group: Period 2006-2015 (ICU survivors) (n=441)**		**OR (95% C.I.)**	**p Value**
	IVDU	0.39 (0.22-0.70)	0.002
	Non-CRBSI	0.25 (0.07-0.87)	0.029
	IMV	0.39 (0.24-0.63)	<0.001
	Surgical wound infection	0.08 (0.02-0.35)	0.001
	RRT	5.74 (1.87-17.66)	0.002

ART: antiretroviral therapy; CI: confidence interval; CRBSI: catheter related bloodstream infection; ICU: intensive care unit; IMV: invasive mechanical ventilation; IVDU: intravenous drug user; OR: odds ratio; RRT: renal replacement therapy.

ST4. Predictors of ICU mortality. Characteristics related to ICU outcomes of the patients included in the study (comparison between survivors and deceased). Univariable analysis

Variables[a]	Total (n=502)	Survivors (n=443)	Deceased (n=59)	OR (95% CI)	p Value
Sex (male)[b]	375 (75%)	326 (74%)	49 (83%)	-*	-*
Age (years)[b]	47.5 (39.7-53.9)	47 (39.7-53.8)	48.3 (39.1-54)	-*	-*
Comorbidities					
Number	0 (0-2)	0 (0-2)	0 (0-2)	0.88 (0.66-1.18)	0.389
Chronic kidney disease	27 (5%)	24 (5%)	3 (5%)	0.96 (0.27-3.46)	0.949
Neoplasm	75 (15%)	64 (14%)	11 (19%)	1.38 (0.67-2.82)	0.383
Liver cirrhosis	116 (23%)	98 (22%)	18 (31%)	1.54 (0.84-2.83)	0.166
Period					
2006-2015	328 (65%)	281 (63%)	47 (80%)	1	**0.020**
2016-2019	174 (35%)	162 (37%)	12 (20%)	0.45 (0.23-0.88)	
Years between HIV diagnosis and hospital admission (n=420)	15.71 (7.41-21.68)	15.72 (7.39-21.56)	15.69 (7.91-22.27)	1 (0.96-1.04)	0.998
Undetectable VL[c] pre-admission (n=403)	257 (64%)	234 (66%)	23 (48%)	0.42 (0.21-0.85)	**0.015**
Pre-admission CD4 count (cells/mm^3) (n=398)	254 (102-468)	290 (121-493)	145.5 (45.5-264)	0.90 (0.83-0.97)[d]	**0.007**
Nadir CD4 count (cells/mm^3) (n=400)	88 (27.5-204.5)	93 (30-205)	64 (24-204)	1 (0.91-1.12)[d]	0.926
Previous OI (n=446)	225 (50%)	191 (48%)	34 (65%)	2.16 (1.16-4.01)	**0.015**
Pre-admission ART (n=445)	367 (82%)	326 (83%)	41 (80%)	0.80 (0.35-1.81)	0.594
Admission source					
Emergency department	299 (60%)	265 (60%)	34 (58%)	1	0.079
Hospital Ward	111 (22%)	92 (21%)	19 (32%)	1.59 (0.86-2.95)	
Others	92 (18%)	86 (19%)	6 (10%)	0.54 (0.22-1.35)	
Days of hospitalization before ICU[e] (n=111)	8 (4-16)	8 (3.5-16)	11 (4-18)	1.02 (0.97-1.06)	0.522
Type of ICU admission (medical/surgical)					
Medical admission	384 (76%)	333 (75%)	51 (86%)	1	0.063
Surgical admission (elective)	76 (15%)	74 (17%)	2 (3%)	0.18 (0.04-0.77)	
Surgical admission (urgent)	42 (8%)	36 (8%)	6 (10%)	1.12 (0.44-2.89)	
Admission due to respiratory infection	162 (32%)	134 (30%)	28 (47%)	2.16 (1.22-3.82)	**0.008**
Admission due to ADE	109 (22%)	89 (20%)	20 (34%)	2.00 (1.10-3.62)	**0.023**
SOFA (admission)	5.5 (3-8)	5 (3-8)	9 (6-13)	1.25 (1.16-1.34)	**<0.001**
APACHE II (admission)	18 (14-22)	18 (14-21)	20 (16-25)	1.08 (1.04-1.13)	**<0.001**
IMV	216 (43%)	164 (37%)	52 (88%)	12.54 (5.56-28.28)	**<0.001**
Vasopressors	220 (44%)	172 (39%)	48 (81%)	6.81 (3.43-13.52)	**<0.001**
RRT	36 (7%)	22 (5%)	14 (24%)	5.76 (2.59-12.81)	**<0.001**
Any ICU therapy (IMV, vasopressors, RRT)	290 (58%)	234 (53%)	56 (95%)	16.51 (5.09-53.58)	**<0.001**
At least 2 ICU therapies (IMV, vasopressors, RRT)	158 (31%)	113 (26%)	45 (76%)	9.36 (4.94-17.76)	**<0.001**
All 3 ICU therapies (IMV, vasopressor, RRT)	24 (5%)	11 (2%)	13 (22%)	10.55 (3.81-29.27)	**<0.001**
Any complication in ICU (n=500)	210 (42%)	169 (38%)	41 (69%)	3.55 (1.95-6.46)	**<0.001**
ART in the ICU (n=429)	228 (53%)	210 (55%)	18 (36%)	0.45 (0.24-0.84)	**0.013**
ICU length of stay (days)	4 (2-9)	4 (2-8)	9 (3-25)	1.03 (1.01-1.05)	**0.001**

[a]Under each variable, the sample size (n) on which the analysis was conducted is provided; [b]Used as adjustment variable; [c]VL: <50 copies/mL; [d]Per increase of 50 cells/mm^{3}; [e]Only for patients admitted from wards; patients coming from emergency department are excluded. * These variables are for adjustment, and their odds ratio (OR) is not interpreted.

Categorical variables are expressed as n (%) and quantitative variables as median (P25-P75). ADE: acquired immunodeficiency syndrome (AIDS)-defining event; APACHE II: Acute Physiology And Chronic Health Evaluation; ART: antiretroviral therapy; CI: confidence interval; HIV: human immunodeficiency virus; ICU: intensive care unit; IMV: invasive mechanical ventilation; OI: opportunistic infection; OR: odds ratio; RRT: renal replacement therapy; SOFA: Sepsis-related Organ Failure Assessment; VL: viral load (copies/mL).

ST5. Predictors of one-year mortality among ICU survivors. Characteristics related to ICU discharge outcomes of the patients included in the study that survived ICU admission (comparison between survivors and deceased). Univariable analysis.

Variables[a]	Total (n = 443)	Survivors (n = 379)	Deceased (n = 64)	OR (95% CI)	P Value
Sex (male)[b]	326 (74%)	274 (72%)	52 (81%)	-*	-*
Age (years)[b]	47 (39.7-53.9)	46.6 (39.3-53.1)	51 (43.95-60.15)	-*	-*
Comorbidities					
Number	0 (0-2)	0 (0-1)	2 (0-2)	1.47 (1.19-1.82)	**<0.001**
Chronic kidney disease	24 (5%)	16 (4%)	8 (12%)	3.33 (1.25-8.85)	**0.016**
Neoplasm	64 (14%)	45 (12%)	19 (30%)	2.80 (1.48-5.27)	**0.001**
Liver cirrhosis	98 (22%)	81 (21%)	17 (27%)	1.29 (0.70-2.39)	0.416
Period					
2006-2015	281 (63%)	237 (63%)	44 (69%)	1	0.137
2016-2019	162 (37%)	142 (37%)	20 (31%)	0.64 (0.36-1.15)	
Years between diagnosis and ICU admission (n=371)	15.72 (7.39-21.56)	15.70 (7.30-21.69)	17.22 (8.60-20.67)	0.98 (0.95-1.01)	0.250
Pre-admission undetectable VL[c] (n=355)	234 (66%)	198 (67%)	36 (62%)	0.60 (0.32-1.12)	0.107
Pre-admission CD4 count (cells/mm^3) (n=350)	290 (121-493)	300 (129-509)	225 (65-410)	0.94 (0.88-1)[d]	0.068
Nadir CD4 count (cells/mm^3) (n=345)	93 (30-205)	100 (34-200)	59.5 (19-212.5)	0.98 (0.85-1.12)[d]	0.751
Previous OI (n=394)	191 (48%)	157 (46%)	34 (61%)	1.98 (1.08-3.63)	**0.028**
Pre-admission ART (n=394)	326 (83%)	271 (81%)	55 (95%)	3.32 (0.98-11.26)	0.054
Admission source					
Emergency department	265 (60%)	237 (63%)	28 (44%)	1	**0.011**
Hospital ward	92 (21%)	71 (19%)	21 (33%)	2.64 (1.40-4.99)	
Others	86 (19%)	71 (19%)	15 (23%)	1.58 (0.76-3.27)	
Days of hospitalization pre-ICU[e] (n=92)	8 (3.5-16)	7 (3-16)	11 (4-22)	1.05 (1.00-1.11)	0.070
Type of ICU admission (medical/surgical)					
Medical admission	333 (75%)	282 (74%)	51 (80%)	1	0.566
Surgical admission (elective)	74 (17%)	65 (17%)	9 (14%)	0.67 (0.30-1.47)	
Surgical admission (urgent)	36 (8%)	32 (8%)	4 (6%)	0.78 (0.26-2.33)	
Admission due to respiratory infection	134 (30%)	114 (30%)	20 (31%)	1.30 (0.71-2.36)	0.393
Admission due to ADE	89 (20%)	74 (20%)	15 (23%)	1.42 (0.76-2.67)	0.276
SOFA (admission)	5 (3-8)	5 (3-7)	7 (4-10)	1.13 (1.05-1.22)	**0.002**
APACHE II (admission)	18 (14-21)	17 (13-21)	21 (16-24.5)	1.07 (1.02-1.11)	**0.002**
IMV	164 (37%)	138 (36%)	26 (41%)	1.22 (0.71-2.10)	0.480
Vasopressors	172 (39%)	147 (39%)	25 (39%)	1.03 (0.60-1.76)	0.921
RRT	22 (5%)	18 (5%)	4 (6%)	1.41 (0.50-4.00)	0.519
Any ICU therapy (IMV, vasopressors, RRT)	234 (53%)	197 (52%)	37 (58%)	1.35 (0.79-2.33)	0.273
At least 2 ICU therapies (IMV, vasopressors, RRT)	113 (26%)	97 (26%)	16 (25%)	0.95 (0.52-1.75)	0.880
All 3 ICU therapies (IMV, vasopressor, RRT)	11 (2%)	9 (2%)	2 (3%)	1.20 (0.30-4.80)	0.795

Any complication in ICU (n=441)	169 (38%)	142 (38%)	27 (43%)	1.27 (0.73-2.21)	0.399
ART during ICU admission	210 (55%)	175 (54%)	35 (66%)	1.49 (0.79-2.80)	0.217
ICU length of stay (days)	4 (2-8)	4 (2-7)	5.5 (3-13.5)	1.03 (1.01-1.05)	**0.001**
ICU readmission during same admission (n=324)	25 (8%)	22 (8%)	3 (6%)	0.68 (0.17-2.68)	0.586
Permanent hemodialysis (n=31)	9 (29%)	8 (31%)	1 (20%)	0.58 (0.06-5.38)	0.630
Home oxygen therapy (n=352)	17 (5%)	16 (5%)	1 (7%)	2.27 (0.39-13.23)	0.361
Discontinued ART at ICU discharge (n=284)	64 (23%)	61 (22%)	3 (27%)	1.33 (0.32-5.54)	0.691
First CD4 count (cells/mm^3) after ICU discharge (n=182)	245 (103-483)	245 (109-490)	273.5 (84-322)	1 (0.99-1)[d]	0.057
CD4 count (cells/mm^3) 3-6 months post-ICU discharge (n=149)	296 (151-464)	296 (152-464)	182 (50-314)	0.99 (0.98-1)[d]	0.127

[a]Under each variable, the sample size (n) on which the analysis was conducted is provided; [b]Used as adjustment variable; [c]VL: <50 copies/mL; [d]Per increase of 50 cells/mm^3; [e]Only for patients admitted from wards; patients coming from emergency department are excluded. * These variables are for adjustment, and their odds ratio (OR) is not interpreted.

Categorical variables are expressed as n (%) and quantitative variables as median (P25-P75). ADE: acquired immunodeficiency syndrome (AIDS)-defining events; APACHE II: Acute Physiology and Chronic Health Evaluation II; ART: antiretroviral therapy; CI: confidence interval; ICU: intensive care unit; IMV: invasive mechanical ventilation; OI: opportunistic infection; OR: odds ratio; 95%; RRT: renal replacement therapy; SOFA: Sepsis-related Organ Failure Assessment; VL: viral load (copies/mL).

References

1. Knaus WA, Draper EA, Wagner DP, et al.: APACHE II: a severity of disease classification system [Internet]. *Crit Care Med* 1985; 13:818–829[cited 2024 Jun 14] Available from: https://pubmed.ncbi.nlm.nih.gov/3928249/

2. Schneider E, Whitmore S, Glynn KM, et al.: Revised surveillance case definitions for HIV infection among adults, adolescents, and children aged <18 months and for HIV infection and AIDS among children aged 18 months to <13 years--United States, 2008 [Internet]. *MMWR Recomm Rep* 2008; 57:1–12[cited 2024 Jun 14] Available from: https://pubmed.ncbi.nlm.nih.gov/19052530/

3. Vincent JL, Moreno R, Takala J, et al.: The SOFA (Sepsis-related Organ Failure Assessment) score to describe organ dysfunction/failure. On behalf of the Working Group on Sepsis-Related Problems of the European Society of Intensive Care Medicine [Internet]. *Intensive Care Med* 1996; 22:707–710[cited 2024 Jun 14] Available from: https://pubmed.ncbi.nlm.nih.gov/8844239/
